# Profile analysis through self-determination theory and intention to be physically active: differences according to gender and age

**DOI:** 10.3389/fpsyg.2023.1277532

**Published:** 2023-10-03

**Authors:** David Manzano-Sánchez

**Affiliations:** Faculty of Education and Psychology, University of Extremadura, Badajoz, Spain

**Keywords:** Physical Education, autonomy support, responsibility, physical activity intention, basic psychological needs, physical activity

## Abstract

**Introduction:**

Physical Education in the current education system has various objectives,including educating students on the values of physical activity and increasing the physical activity levels of students.

**Objective:**

The purpose of the present study is to analyze the motivational profiles of students, to understand which profiles have higher levels of responsibility, satisfaction of autonomy, competence, and social relationship needs; intention to be physically active; and perception of autonomy support.

**Methods:**

A total of 752 students from Primary, Secondary, and Non-compulsory Education (M = 13.809; SD = 1.984, 47.9% boys and 52.1% girls), from different educational centers in Spain, participated in the study, to whom a series of questionnaires were administered to find out their values of the mentioned variables. The results established the existence of four profiles: “high quality,” “low quality,” “high quantity,” and “low quantity” of motivation.

**Results:**

The results reflect that the students of the “high quality” and “high quantity” profiles had higher values in all the variables in relation to the other two groups (except in amotivation and external regulation), discussing the differential analysis between the four groups. The group with the best results was the “high quantity” profile, as opposed to the “low quantity” profile. In turn, no differences were found according to gender, but according to the educational stage, the Primary Education stage was more related to the more self-determined profiles.

**Discussion and conclusion:**

Therefore, it is necessary to look for more self-determined motivational profiles from an early age in order to improve levels of responsibility, perception of autonomy, satisfaction of basic psychological needs, and the intention to be physically active.

## 1. Introduction

Physical activity plays a crucial role in the overall well-being and development of young people. Numerous studies have highlighted the positive impact of regular exercise on various aspects of adolescent health. According to Meng et al. (2022), physical activity in students can improve body composition, cardiorespiratory fitness, and cardiometabolic markers. Additionally, research conducted by Janssen and Leblanc (2010) highlighted the association between physical activity and enhanced mental health, including reduced symptoms of anxiety and depression. Moreover, findings from a meta-analysis by Eime et al. (2013) revealed that participation in sports and physical activities positively influences social interactions and fosters a sense of belonging in young people. These findings collectively emphasize the importance of promoting and encouraging physical activity among young people to ensure their holistic development and well-being.

In this sense, promoting physical activity through education is of paramount importance in fostering a healthier and more active society. As pointed out by Tremblay et al. (2014), incorporating physical activity into the educational curriculum not only improves students’ overall health but also positively impacts their cognitive and academic performance. This synergy between education and physical activity is further supported by the findings of Day et al. (2019), who wrote that well-designed educational programs can effectively influence behavior change and create lasting habits that promote a physically active lifestyle.

On the other hand, motivation can be defined as the internal drive and enthusiasm that initiates, guides, and sustains goal-directed behavior. It is the force that compels individuals to act in pursuit of their desires and objectives, pushing them to overcome obstacles and persevere in the face of challenges. The Theory of Self-determination (SDT), proposed by Deci and Ryan (1985), is a crucial framework for understanding human motivation and its significance in various domains. According to Deci and Ryan (2000), the theory posits that individuals have innate psychological needs (BPN): autonomy, which reflects the possibility of carrying out activities by one’s own choice (Reeve, 2006); competence, involving a desire to achieve satisfactory results (Deci and Ryan, 1985); and relatedness, which refers to having a good social relationship with others, i.e., a reciprocal relationship (Leyton-Roman et al., 2020), which is essential for fostering intrinsic motivation. This theory explains that motivation goes from more autonomous and self-determined states of motivation (intrinsic motivation), continuing through identified motivation and introjected regulation, until it reaches more external motivation (extrinsic motivation) and, finally, lack of motivation (amotivation). In this field, White et al. (2021) focused on analyzing SDT, explaining that this theory is organized along a continuum involving the level of self-determination of a subject (i.e., the degree to which behavior is performed voluntarily, especially in intrinsic and identified motivation). Furthermore, Vallerand et al. (1992) said that intrinsic motivation plays a pivotal role in driving individuals to engage in activities for the inherent enjoyment and satisfaction they bring, leading to better performance and overall well-being. In educational settings, teachers who support students’ autonomy and provide opportunities for skill development and interpersonal connections can foster a sense of intrinsic motivation (Vansteenkiste et al., 2009), ultimately enhancing learning outcomes and promoting a positive learning environment.

Motivation has been widely studied by educational researchers. For instance, the authors of the SDT theory, Deci and Ryan (2017), investigated the significance of intrinsic motivation in students’ learning experiences and revealed that when learners are intrinsically motivated, they exhibit a genuine interest in the subject matter, leading to enhanced comprehension and long-term retention. At the same time, Physical Education plays a crucial role in fostering motivation (Moy et al., 2016) among students. In brief, SDT is a social cognitive theory that helps to explain student motivation in the context of PE classes (Deci and Ryan, 2000; Leyton-Roman et al., 2020).

The results of promoting motivation and autonomy in Physical Education classes facilitate the enhanced responsibility of students (Pozo et al., 2018; Valero-Valenzuela et al., 2019), autonomy support perception (Chang et al., 2016; Gil-Arias et al., 2020), or physical activity intention (Leyton-Roman et al., 2020).

In this line BPNs are determined by the social environment and autonomy support. In this sense, autonomy support consists of giving students the opportunity to make their own decisions (Reeve, 2016) and this variable is one of the most studied factors in the academic context. In this field, the relationship between autonomy support and BPN has been widely studied by different authors (Aguirre et al., 2016; Pérez-González et al., 2019; Gil-Arias et al., 2020; McCurdy et al., 2020) and this autonomy support is known as a facilitator of students’ autonomous behaviors (Borg and Alshumaimeri, 2019).

On the other hand, physical activity intention is a variable that predicts physical activity (Camacho-Miñano et al., 2013; Moreno-Murcia et al., 2018; Cid et al., 2019). Physical activity intention is important to reduce sedentary behaviors (Biswas et al., 2015; Edwardson et al., 2020) and improve physical activity during free time and self-determined motivation (Franco et al., 2017); it also has other benefits like improved memory, attention, information processing levels (Hillman et al., 2014), and self-esteem (Singh et al., 2015).

Responsibility is becoming an increasingly important phenomenon (Bugdayci, 2019) and is defined as making selections and accepting the consequences and effects of these selections, including taking care of oneself and others, fulfilling our obligations, and participating in society (Lickona, 1991). There are numerous recent studies based on the importance of increasing responsibility in students. For instance, the review by Shen et al. (2022) provides recommendations for responsibility programs, including collaborative efforts focusing on the importance of developing responsibility for students. This study includes 41 high-quality articles on the use of a Personal and Social Responsibility Model, whose main purpose is to promote responsibility in students, and many of these studies are linked to the SDT (Manzano-Sánchez and Valero-Valenzuela, 2019; Merino-Barrero et al., 2019).

However, there are a number of gender differences in motivation according to the meta-analysis of Turhan (2020). Usually, girls are more likely to be motivated by intrinsic factors, such as personal interests or helping others, and they have more academic motivation, while boys are more likely to be motivated by extrinsic factors, such as rewards and recognition, and their academic motivation is lower than girls (Bugler et al., 2015). In line with this, physical activity is lower in girls than in boys, but the most important gap in the literature is that motivation for physical activity support and the premise of self-determined motivation are strongly linked to higher physical activity participation (Lauderdale et al., 2015). This conclusion is in line with that of Shen (2015), who said that boys had higher intrinsic motivation and teacher autonomy support. The same conclusion was achieved by Abdoshahi et al. (2022) in a study involving primary school students, where the boys had higher scores in perceived autonomy support, intrinsic motivation, and intention to physically perform activities.

In the same field, it is important to study the age of participants because of the influence of educational stage on the academic motivation of girls and boys (Turhan, 2020); for this reason, studying both together is highly important. According to age, Nigg and Amarto (2015) indicate that the habits that are acquired during infancy and Primary school have a positive or negative impact on adolescence and in the future. Thus, the transition from primary to Secondary Education has been described as a phase of psychological, biological, and emotional transformation typical of entry into adolescence (Prieto and Delgado, 2017).

The secondary stage is considered a period of great difficulties due to the lack of motivation toward studies (Martínez and Blanco, 2005). In this sense, Manzano-Sánchez (2021) said that secondary school students have worse values in relation to motivation, BPNs, and responsibility than primary school students. Physical activity according to Singerland et al. (2011) is lower in secondary school students than primary students, especially in girls; furthermore, secondary school boys were found to be more active than girls. However, Jago et al. (2012) indicated that boys’ after-school physical activity declined by 16% after the move from primary to secondary school, compared to a 12% decline for girls. This is not a conclusion regarding whether the reduction of physical activity is higher in boys or girls, but there is evidently a reduction when students progress to the Secondary Education stage.

The purpose of this study is to study the motivational profiles of secondary and primary students to identify the differences in physical activity intention, autonomy support, and responsibility, identifying the differences between gender and educational stage. We hypothesized that (1) there would be different motivational profiles following the theory of Deci and Ryan and different studies (Yli-Piipari et al., 2009; Haerens et al., 2010; Sánchez-Oliva et al., 2015), (2) primary students would be more likely to be in the “high quality” or “high quantity” profiles than secondary and non-compulsory school students (Manzano-Sánchez, 2021), and (3) boys would have higher intention to be physically active and higher intrinsic motivation than girls, especially in the secondary stage (Bugler et al., 2015; Turhan, 2020; Aznar-Ballesta and Vernetta, 2023).

## 2. Method

### 2.1. Procedure

This is a cross-sectional and quantitative study. The questionnaires were coded on the online survey platform Google Forms^1^ and dates were collected from February to May 2023. First, contact was made with the different participating centers, through well-known Physical Education teachers, having different meetings with the corresponding management teams via Zoom or in person from different centers of Spain, specifically, Murcia (Región de Murcia), Alicante (Comunidad Valenciana), and Toledo (Castilla La Mancha). The link to the questionnaire was sent to Physical Education teachers, and a Zoom meeting was carried out to explain how to pass the survey to the students, this meeting lasted between 20 and 25 min. This questionnaire started with a presentation of the study, informing the participants of the objectives, including a clause of confidentiality of the data, where the participants had to indicate in the first place that they agreed to participate in the study and that they had understood the indicated information. After that, they answered sociodemographic questions and completed different questionnaires about motivation, physical activity intention, responsibility, and autonomy support. The time to complete the questionnaire was approximately 20–25 min. All procedures that were carried out were in accordance with the standards of the Helsinki Declaration and were approved by the University of Murcia Ethical Committee (1,685/2017).

### 2.2. Participants

This study adopted accessibility and convenience sampling selection. A total of 775 questionnaires were recovered, and after statistical atypical case selection with Mahalanobis Distance and according to exclusion criteria (one answer per participant and an answer to all questions), the final sample consisted of 752 students (97.03%, M = 13.675; SD = 1.967) from three Spanish regions: Comunidad Valenciana Region de Murcia and Castilla-La Mancha. The sample consisted of 360 boys (47.9%) and 392 girls (52.1%). Following the Spanish Education System, the students were from Primary and Secondary Education. Specifically, 253 (33.6%) from Primary Education (year 4 to year 6); Secondary Education, from year 7 to year 10 (61.3%); and 38 from non-compulsory education (5.1%).

### 2.3. Measures

#### 2.3.1. Academic motivation

A Motivation in Physical Education Questionnaire was used [CMEF, Sánchez-Oliva et al. (2012)]. This questionnaire is composed of 20 items. The items are established on a Likert scale from totally disagree (1) to totally agree (5). This questionnaire includes four items for each scale. This questionnaire is composed by five scales. Specifically, intrinsic motivation (“Because Physical Education is fun”), identified motivation (“because I value the benefits that this subject can have on my self-development”), introjected regulation (“because it’s what I have to do to feel good”), external regulation (“because it is approved by the teacher and the classmates”), and amotivation (“I do not understand why we should have Physical Education”). The alpha’s Cronbach values were α = 0.867 (intrinsic motivation), α = 0.866 (identified motivation), α = 0.700 (introjected regulation), α = 0.781 (external regulation), and α = 0.702 (amotivation).

#### 2.3.2. Satisfaction of basic psychological needs

Basic Psychological Needs Satisfaction Questionnaire [BPNES by Vlachopoulos and Michailidou (2006)]: to measure the satisfaction of basic psychological needs (BPNs). A Spanish version from Moreno-Murcia et al. (2008) was used. This scale has 12 items that aim to investigate autonomy values (“the types of exercise I do are in line with my interests”), competence (“exercising is something I do very well”), and relationship (“I feel very comfortable with my colleagues”). This questionnaire has a Likert-type scale from 1 (totally disagree) to 5 (totally agree). The Cronbach’s alpha values obtained were α = 0.842 (autonomy), α = 0.818 (competence), and α = 0.866 (relationship).

#### 2.3.3. Responsibility

A Personal and Social Responsibility Scale [PSRQ by Li et al. (2008)] was used with the Spanish version developed by Escartí et al. (2011). This questionnaire is composed of two scales (personal responsibility and social responsibility) with a total of fourteen items and a Likert-type scale ranging from totally agree (1) to totally disagree (6). The internal consistency was α = 0.841 for personal responsibility and α = 0.904 in the case of social responsibility.

#### 2.3.4. Autonomy support

An Autonomy Support Scale (EAA-EF, Moreno-Murcia et al., 2020) was used to check the perception of teacher support by students. This questionnaire has 11 items and a Likert scale with five responses, ranging from definitely not (1) to definitely yes (5). An example of an item is “They value our ideas and suggestions and let us propose things.” The internal consistency value was α = 0.828.

#### 2.3.5. Physical activity intention

Measurement of the Intention to be Physically Active [MIFA in Spanish by Moreno-Murcia et al. (2008) adapted from Hein et al., (2004)] was used to analyze the physical activity intention of the participants. This scale is composed of five items on a Likert scale from 1 “strongly disagree” to 5 “strongly agree.” An example of an item is “I usually practice sports in my free time.” The internal consistency value was α = 0.811.

### 2.4. Statistical analysis

First, the database was filtered by applying the Mahalanobis distance once the data from the questionnaires had been entered, this distance was applied considering the variables that were built for the clusters (intrinsic motivation, identified motivation, introjected regulation, external regulation, and amotivation). Next, we calculated the mean and standard deviation for the scores and the data were Z-transformed to be standardized. We also investigated the correlation between variables, and the values of skewness and kurtosis were used to check the normality, considering values <3 and < 7, respectively, as normal values (Curran et al., 1996) and < 1.98 following Field (2017). After that, the Cronbach’s alpha coefficient was calculated to check the reliability of each variable. All variables that had values over 0.70 were considered acceptable (Viladrich et al., 2017).

Then, we checked the student’s profiles in a two-step cluster analysis approach using a combination of hierarchical and non-hierarchical methods (Hair et al., 2018). Subsequently, a hierarchical conglomerate analysis was performed using Ward’s method (Euclidean distance square) with Z-standardized scores of intrinsic motivation, identified motivation, introjected regulation, external regulation, and amotivation. We checked the dendrogram with a distance between 5 and 10 points, and a four-cluster solution was found to be the most suitable, so we selected this solution.

Furthermore, a univariate analysis of variance was performed to check the explanatory power of the cluster solution. In addition, we carried out a double-split cross-validation approach (the sample was randomly split into halves, and the same procedure was then repeated). The degree of agreement with cluster solution was 0.61 (*p* = 0.001) with the Cohen’s kappa test. This is a value that is considered appropriate according to Breckenridge (2000).

In order to check the differences in the variables of BPNs, responsibility, physical activity intention, and autonomy support, a multivariate analysis of variance was performed, including *F* value and size effect. A *post hoc* contrast was used with the Bonferroni test to check the differences between profiles. Size effect was considered, following Richardson (2011), as small (< 0.01), medium (0.01 to 0.06), medium-large (0.06 to 0.14), or large (>0.14). Furthermore, we examined the differences in gender and educational stage within each subgroup by checking the differences in the distribution in the different profiles and the statistical differences. All analysis was performed with IBM SPSS, v. 25.0 (SSPS Inc. Chicago IL, EE.UU) establishing the level of significance *p* < 0.05.

## 3. Results

### 3.1. Descriptive and correlation results

Table 1 shows the descriptive results of the different variables under study. The correlation between the variables was positive in all cases and significant (*p* < 0.01), except for amotivation, which was negative (except for the autonomy and autonomy support variables, where it was not significant). It is noteworthy that the highest correlation happened between intrinsic motivation and the three BPNs. At the same time, the BPN of competence had the highest correlations with responsibility and intention to be physically active than autonomy and relation. In turn, the skewness and kurtosis values were checked, showing adequate values (<2) in any case, as indicated in the statistical analysis section.

**Table 1:** Descriptive analysis and correlations.

		Mean	*SD*	*R*	*S*	*K*	2	3	4	5	6	7	8	9	10	11	12
1	Intrinsic M.	3.90	1.00	1–7	−0.900	0.174	0.780**	0.516**	0.358**	−0.216**	0.626**	0.680**	0.625**	0.540**	0.343**	0.383**	0.553**
2	Identified M.	3.65	1.04	1–5	−0.575	−0.432	1	0.534**	0.365**	−0.177**	0.643**	0.618**	0.560**	0.567**	0.343**	0.394**	0.497**
3	Introjected R.	3.05	1.02	1–5	−0.036	−0.661		1	0.585**	0.080*	0.485**	0.445**	0.364**	0.359**	0.227**	0.257**	0.343**
4	External R.	2.87	1.10	1–5	0.141	−0.869			1	0.215**	0.371**	0.320**	0.237**	0.311**	0.180**	0.176**	0.200**
5	Amotivation	1.63	0.82	1–5	1.312	0.966				1	−0.048	−0.138**	−0.154**	−0.059	−0.160**	−0.253**	−0.145**
6	Autonomy	3.12	1.03	1–5	−0.824	−0.057					1	0.660**	0.594**	0.639**	0.355**	0.348**	0.439**
7	Competence	3.70	0.95	1–5	−0.121	−0.701						1	0.693**	0.504**	0.408**	0.451**	0.615**
8	Relation	3.84	1.03	1–5	−0.584	−0.325							1	0.480**	0.444**	0.371**	0.490**
9	Autonomy S.	3.66	0.95	1–5	−0.797	−0.168								1	0.362**	0.323**	0.366**
10	RPS	4.96	0.98	1–6	−0.667	−0.145									1	0.707**	0.357**
11	RPP	5.02	0.96	1–6	−1.295	1.646										1	0.422**
12	MIFA	4.00	0.90	1–6	−1.318	1.511											1

M, Mean; SD, Standard deviation; R, Range; S, Skewness; K, Kurtosis; M, motivation; R, Regulation; S., Support; RPS, Social responsibility; RPP, Personal responsibility; MIFA, Physical activity intention = *p* < 0.01; * = *P* < 0.05.

### 3.2. Cluster profile result

**Figure 1:**
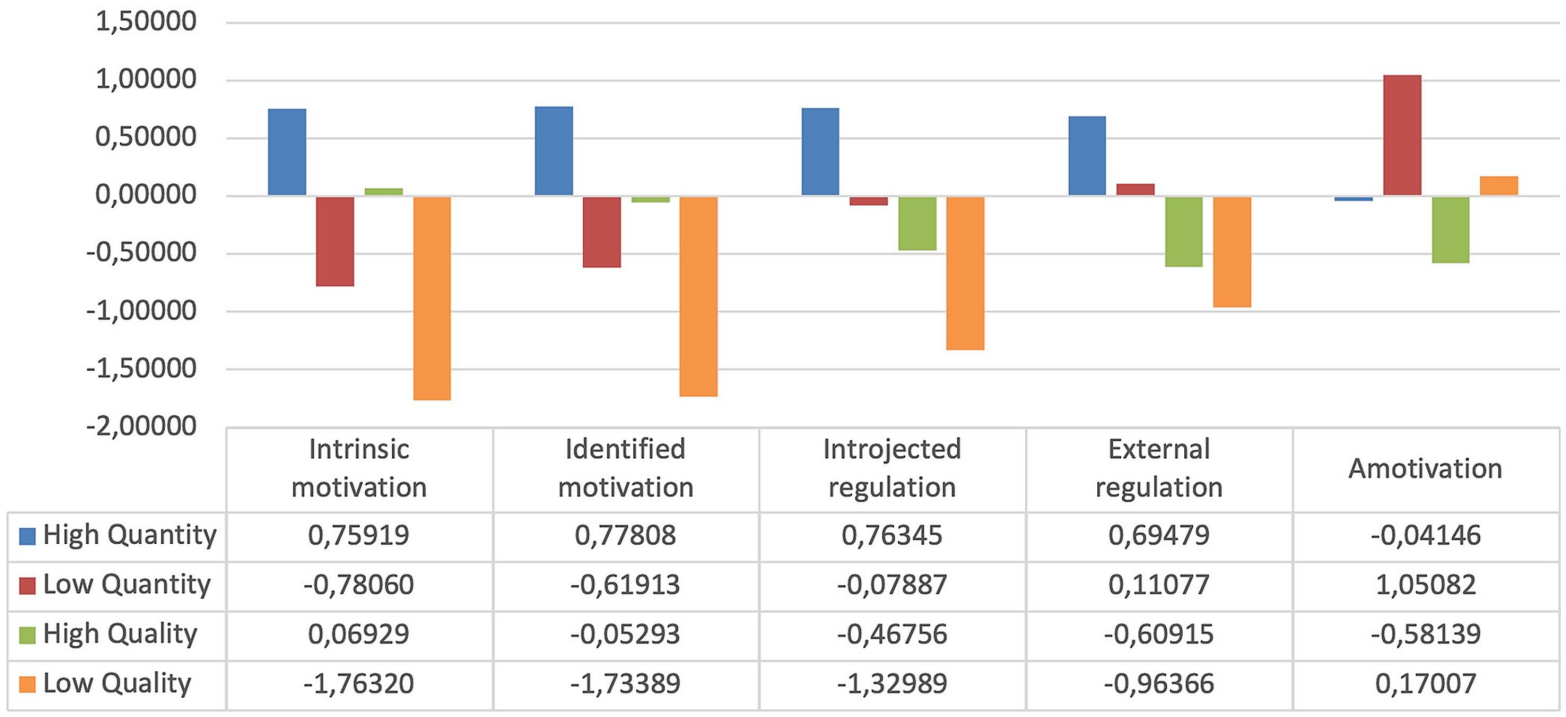
Motivational profiles.

With the final 752 participants (after exclusion criteria were applied), we started with cluster analysis. The dendrogram and the agglomeration coefficients reflected that the most adequate solution would be four or six profiles. Finally, we selected the four solutions due to the coefficients being increased highly by the movement between these two profiles, and the four-cluster solution has been supported in previous research (Sánchez-Oliva et al., 2015; Manzano-Sánchez et al., 2021). Finally, we checked the four-cluster solution and found that it was the profile that explained the variance of clustering of 68.2% (*R*_2_ = 0.682; *R* = 0.832). This cluster had significant correlations in *p* < 0.001 for intrinsic motivation, identified motivation, introjected regulation, external regulation, and amotivation. In Figure 1, we can see the values of the four profiles.

**Figure 2:**
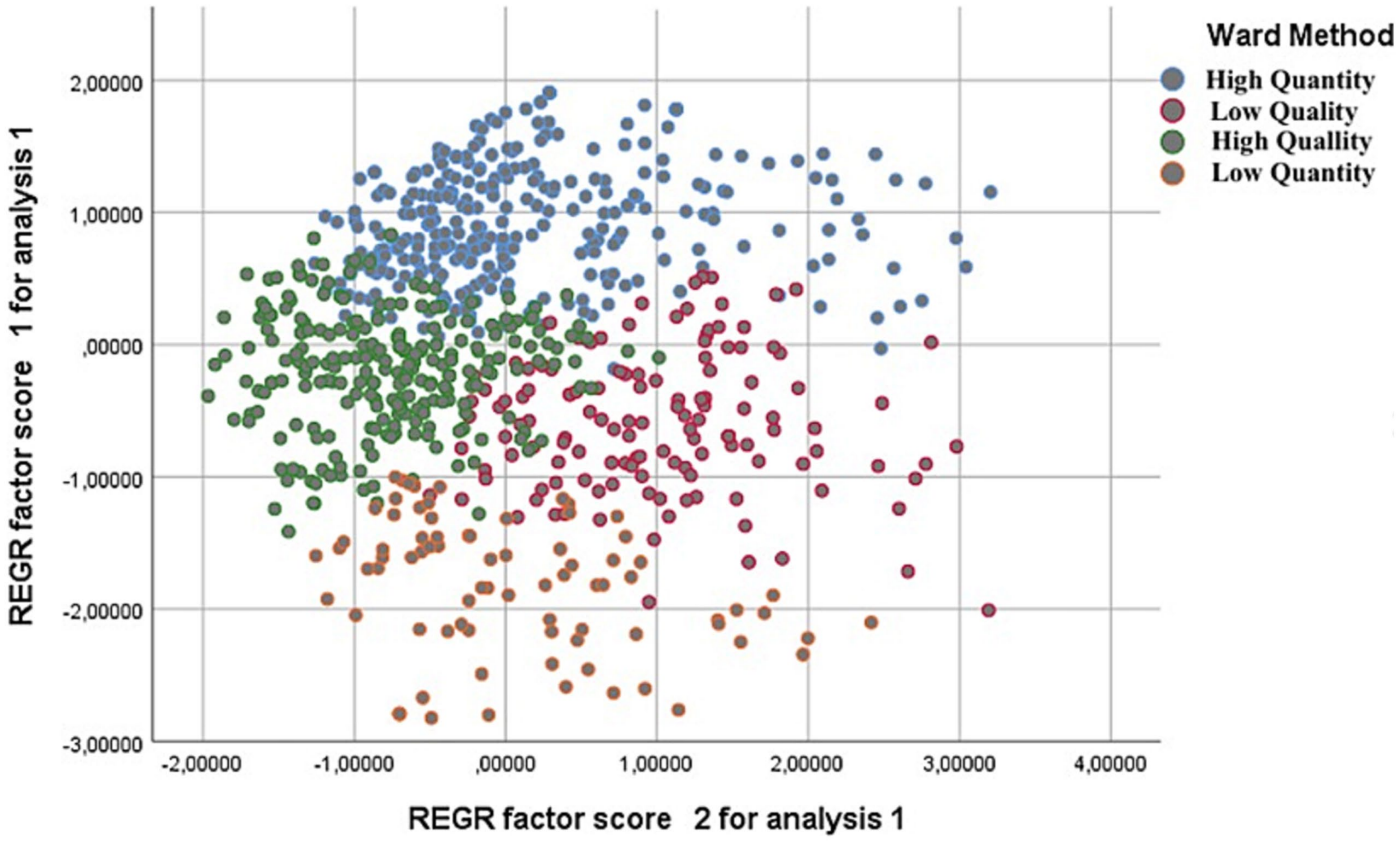
Dispersion diagram from cluster. Y-axis, Autonomous motivation; X-axis, Controlled motivation and amotivation.

The non-hierarchical cluster confirmed the four-cluster solution. The profiles were named “high quantity” (n = 301, 40.0%), with high values in all autonomous and external motivation; “low quality,” with high values in amotivation and low values in more internal motivation (*n* = 131; 17.4%); “high quality,” with low levels of external motivation and amotiation and positive values in identified and intrinsic motivation (*n* = 239; 31.8); and “low quality,” with very low values of motivation (internal and external) and high levels of amotivation (*n* = 81; 10.8%). On the other hand, in Figure 2, we can see a scatter plot where the Y-axis is autonomous motivation and the X-axis is controlled and amotivation. We can see that the majority of the participants have values on the X-axis from 0.000 to 3.000 in the “low quality” and “low quantity” profiles. On the other hand, the majority of the other two profiles are between −1.000 and 2.000 on the Y-axis (Figure 2).

In Table 2, we can check the differences between the motivation variables from each cluster. The multivariate effect was significant at *p* < 0.001, pointing to the violation of the assumption of homogeneity. In this sense, the “high quantity” profile has greater values of all kinds of motivation. On the other hand, the “low quality” profile was the profile with more amotivation values and reduced values in internal and external motivation. The “high quality” profile had lower values in amotivation and high levels in intrinsic and identified motivation, with intermediate values in extrinsic regulation and introjected motivation. Finally, the “low quantity” profile had lower values in all variables except amotivation, for which it had the second highest values.

**Table 2:** Profile analysis according to motivation.

	Hiqh quantity		Low quality		High quality		Low quantity		*F*	*p*	eTa
	*M*	SD	*M*	SD	*M*	SD	*M*	SD			
Intrinsic motivation	4.66	0.40	3.11	0.71	3.97	0.61	2.13	0.73	515.813	0.000***	0.674
Identified motivation	4.46	0.47	3.01	0.73	3.60	0.76	1.85	0.54	433.140	0.000***	0.635
Introjected regulation	3.83	0.74	2.97	0.65	2.57	0.69	1.69	0.56	244.141	0.000***	0.495
External regulation	3.64	0.93	2.99	0.75	2.2	0.86	1.81	0.57	176,056	0.000***	0.414
Amotivation	1.60	0.87	2.50	0.67	1.16	0.29	1.77	0.80	108.809	0.000***	0.304

*M* de box = 551.441, f = 12.061, *p* = < 0.001; Pillai trace = 1.210 f (100.855), *p* < 0.01.

*M*, Mean; SD, Standard deviation; eTa, size effect; F, f-test from Multivariate analysis (MANOVA); *** *p* < 0.001.

Finally, according to one of the main purposes of this study, we investigated the values of responsibility, BPNs, autonomy support, and physical activity intention between the profiles (Table 3). In order to know which groups were different from each other, we used the Bonferroni-correction test.

**Table 3:** Profile analysis according to BPNs, responsibility, autonomy support, and physical activity intention.

	High quantity		Low quality		High quality		Low quantity		F	*p*	eTa
	M	SD	M	SD	M	SD	M	SD			
Autonomy	3.75	0.79	2.65	0.87	2.99	0.89	1.91	0.75	130.940	0.000**	0.344
Competence	4.28	0.60	3.13	0.84	3.71	0.80	2.47	0.86	160.497	0.000**	0.392
Relatedness	4.38	0.69	3.22	1.01	3.87	0.90	2.71	1.00	110.563	0.000**	0.207
RPP	5.29	0.73	4.51	1.08	5.01	0.88	4.31	1.32	37.101	0.000**	0.130
RPSS	5.34	0.65	4.59	1.09	5.14	0.83	4.20	1.26	48.435	0.000**	0.163
Autonomy_Support	4.15	0.66	3.28	0.90	3.58	0.86	2.72	1.15	78.985	0.000**	0.241
MIFA	4.44	0.61	3.43	0.85	4.05	0.76	3.13	1.14	90.650	0.000**	0.267

*M* de box = 420.376, f = 4.595, *p* = < 0.001.

Pillai trace = 0.258 *f* (22.719), *p* = < 0.001.

M, Mean; SD, Standard deviation; RPP, Personal responsibility; RPSS, Social responsibility; MIFA, Physical activity intention; eTa, size effect; F, *f*-value from Multivariate analysis (MANOVA); **p < 0.001.

### 3.3. Differences in motivational profile between groups

The four motivational profiles identified significantly differed from one another with respect to BPNs, personal and social responsibility, autonomy support, and physical activity intention. The results, using multiple comparisons, contrasted with Bonferroni’s correction, are in Table 4. *Post hoc* analysis reported that, in the case of autonomy, all variables had significant differences between clusters in *p* < 0.01 or *p* < 0.001 in favor of the “high quality” and “high quantity” profiles. Competence and relatedness followed a similar line, with higher values in the “high quality” and “high quantity” profiles. On the other hand, in personal and social responsibility, significant differences were found between the four motivational profiles, except between the “low quality” (2) and “low quantity” (4) profiles, with the highest values being recorded for the “high quality” profile, followed by the “high quantity” profile. Autonomy support had differences between profiles, with higher values in the “high quality” profile, followed by the “high quantity,” “low quality,” and finally, “low quantity” profiles.

**Table 4:** Analysis between clusters.

	1 vs. 2	1 vs. 3	1 vs. 4	2 vs. 3	2 vs. 4	3 vs. 4
Autonomy	1.97***	0.765***	1.844***	−0.331**	0.747***	1.079***
Competence	1.153***	0.565***	1.807***	−0.587***	0.654***	1.241***
Relatedness	1.160***	0.511***	1.677***	−0.649***	0.516***	1.165***
RPP	0.747***	0.202*	1.14***	−0.545***	0.389*	0.934***
RPSS	0.781***	0.284**	0.981***	−0.497***	0.200	0.697***
Autonomy_Support	0.868***	0.577***	1.431***	−0.292**	0.563***	0.855***
MIFA	1.006***	0.387***	1.308***	−0.619***	0.301*	0.920***

M, Mean; SD, Standard deviation; RPP, Personal responsibility; RPSS, Social responsibility; MIFA, Physical activity intention; eTa, size effect; F, *f*-test from Multivariate analysis (MANOVA); * *p* < 0.05; ** *p* < 0.001; *** *p* < 0.001; 1, “High quantity”; 2, “Low quality”; 3, “High quality”; 4, “Low quantity”.

Finally, physical activity intention was higher in the “high quality” profile and the “high quantity” profile, with lower values being recorded for the “low quality” profile, and lower still for the “low quantity” profile. The only variable that did not have differences between the groups was social responsibility (between high and low quality and low quantity) and the significance of personal responsibility and MIFA for these groups was low (near to 0.50).

### 3.4. Differences between profiles according to gender and educational stage

Following Manzano-Sánchez et al. (2021), to check the differences in the distribution of the motivational profiles found in terms of gender and the course, it was decided that a difference analysis using Pearson’s chi-square statistic with cross tables would be performed. This test is adequate for observed and expected frequencies in a category to test whether all categories contain the same or different proportions of values for a user-specified proportion. We used corrected typified residuals to provide us with information regarding where the differences were found (greater >1.90 indicated that differences were significant).

In this sense, we did not find any differences between genders in the group distribution; however, the educational stage was found to be related to the different profiles. In summary, primary school students were in the “high quantity” profile, and this group was the most adequate in motivation, responsibility, satisfaction of BPNs, autonomy support, and physical activity intention. On the other hand, Secondary students were the group with the second highest number of students in the “high quantity profile” and the group with the most students in the “high quality” profile (All these variables were greater than 1.9). Finally, Non-compulsory Education students had a similar distribution in their profiles, and the only difference was in the low quantity profile, with a standardized residual of 2.1 (21.1% of the participants) (Table 5).

**Table 5:** Differences according to gender and educational stage.

	High quantity			Low quality			High quality			Low quantity			X^2^	gl	*p*
	*n*	%	R	*n*	%	R	*n*	%	R	*n*	%	R			
Men	144	19.1%	0.0	64	8.5%	0.2	113	15.0%	0.2	9	1.2%	0.1	0.87	3	0.993
Girl	157	20.9%	0.0	67	8.9%	0.2	126	16.8%	0.2	64	8.5%	0.1			
P. School	146	57.7%	7.0	26	10.7%	3.5	71	28.1%	−1.6	9	3.6%	−4.5	64.544	6	0.000**
S. School	144	31.2%	6.2	94	20.4%	2.7	159	34.5%	2.0	64	13.9%	3.5			
NC School	11	28.9%	1.4	20	23.3%	1.5	9	23.7%	−1.1	8	21.1%	2.1			

P, Primary; S, Secondary; NC, Non-compulsory; R, Standardized Residual, SD, Standard Deviation; PBN, Basic Psychological Needs; X2, chi squared; gl, free grades.

### 3.5. Differences according to gender and educational stage

Finally, we investigated the differences according to gender and educational stage. Gender did not have any differences in any variables, with similar values between boys and girls, taking into account the sample in general (Table 6).

**Table 6:** Gender and stage differences.

	Boys		Girls		F	*p*	eTa	Primary Education		Secondary Education		Non-compulsory Education		F	*p*	eTa
	*M*	SD	*M*	SD				*M*	SD	*M*	SD	*M*	SD			
Intrinsic motivation	3.88	0.98	3.91	1.02	0.264	0.609	0.000	4.31	0.81	3.70	1.02	3.49	1.13	36.270	0.000**	0.088
Identified motivation	3.61	1.02	3.69	1.06	1.443	0.248	0.002	4.07	0.86	3.47	1.05	3.10	1.07	36.775	0.000**	0.089
Introjected regulation	3.10	1.00	3.00	1.04	1.953	0.172	0.002	3.38	0.97	2.89	1.01	2.78	1.03	21.019	0.000**	0.053
External regulation	2.90	1.05	2.85	1.14	0.569	0.492	0.001	3.14	1.14	2.74	1.06	2.71	1.01	11.666	0.000**	0.030
Amotivation	1.65	0.85	1.62	0.80	0.282	0.519	0.001	1.63	0.84	1.63	0.81	1.70	0.81	0.120	0.888	0.000
MIFA	4.02	0.87	3.98	0.93	0.349	0.512	0.000	4.21	0.77	3.90	0.93	3.77	1.08	10.733	0.000**	0.035
Autonomy	3.08	1.01	3.15	1.04	0.920	0.372	0.001	3.44	0.93	2.96	1.04	2.90	0.93	19.431	0.000**	0.049
Competence	3.74	0.93	3.67	0.97	0.797	0.920	0.001	4.00	0.87	3.58	0.95	3.26	1.05	21.901	0.000**	0.055
Relatedness	3.84	1.02	3.84	1.03	0.002	0.962	0.000	4.22	0.84	3.67	1.05	3.42	1.14	28.834	0.000**	0.071
Autonomy_Support	3.61	0.96	3.71	0.95	2.009	0.138	0.003	3.90	0.75	3.56	1.02	3.32	1.05	13.414	0.000**	0.035
Social_Responsibility	4.91	1.01	5.01	0.96	1.912	0.167	0.003	5.26	0.84	4.79	1.00	4.98	1.20	18.884	0.000**	0.048
Personal_Responsibility	4.98	0.97	5.06	0.95	1.173	0.279	0.002	5.33	0.70	4.87	1.03	4.85	1.06	20.751	0.000**	0.053

*M* de box = 66.766, f = 0.867, p = 0.793.793.

Pillai trace = 0.022, *f* (1.388), *p* = 0.166.

M, Mean; SD, Standard deviation; MIFA, Physical activity intention eTa, size effect; F, *f*-test from Multivariate analysis (MANOVA), *** *p* < 0.001.

However, Table 6 shows the differences between stages, especially in variables with *p* < 0.001, except in amotivation, where differences did not occur between groups. Primary Education is a stage where the students have higher levels of internal and external motivation to engage in physical activity, and at the same time, they have good satisfaction with their BPNs (all these variables are higher in this group compared to Secondary Education and Non-compulsory Education). On the other hand, they feel that their teacher provides them with a good environment to be autonomous and they feel high levels of social and personal responsibility.

The differences between Secondary Education and Non-compulsory Education are in intrinsic motivation and identified motivation, physical activity intention, relatedness and competence, and autonomy support (higher in Secondary Education) following the post-hoc test. However, Non-compulsory students had higher levels of social responsibility.

## 4. Discussion

The purpose of this study is to study the motivational profiles of secondary and primary school students to identify the differences in physical activity intention, autonomy support, and responsibility and show the differences between gender and educational stage. The hypothesis was that (1) there would be four motivational profiles; (2) primary students would be more likely to be in the “high quality” or “high quantity” profiles than Secondary and Non-compulsory school students; and (3) boys would have higher intention to be active and have greater intrinsic motivation than girls, especially in the secondary stage.

According to the first hypothesis, we can confirm that there were four profiles, as initially hypothesized, agreeing with the studies by Sánchez-Oliva et al. (2015), where they found four profiles with 1,690 Secondary Education students, and Manzano-Sánchez et al. (2021), with 768 participants. However, in the studies cited, the levels of amotivation were also high in the profile called “high quantity,” which possibly caused this profile to have fewer adaptive consequences than the “high quality” profile, indicating to the authors that it could be due to a “standardized response” from the participants, which was not the case in the present study. In our study, on the other hand, the “high quantity” profile proved to be the one with the most positive results. This indicates that high levels of internal and external motivation (not amotivation) could play a significant role in improving adherence to physical activity, improving responsibility, satisfying BPNs, and the perception of autonomy support. Finally, note that the solution of profiles is an area still under study since other studies have identified the existence of two profiles (Yli-Piipari et al., 2009) or even five profiles (Haerens et al., 2010), probably due to the use of different motivational variables like autonomous or controlled motivation or the self-determination index. In this sense, future studies have to consider the amotivation variable, since it could have a negative influence on a “high quantity” profile.

Secondly, based on the second hypothesis, we must highlight that Romera et al. (2022) evidenced the role of age and gender in different adolescent behaviors and that, usually, girls would have higher levels of social values and less disruptive behaviors. Following this author, preadolescence (primary school) would be a particularly relevant stage to educate and develop values and social rules adjusted to the context. Our study corroborates that the educational stage of students has a special relevance to motivational factors. In this way, the Primary Education stage had the highest percentage of students in the “high quantity” and “high quality” profiles, which translated into higher levels of intention to be physically active, satisfaction of BPNs, responsibility, and autonomy support. All of this generates the need for Physical Education to seek to improve motivation and adherence to physical activity outside and inside school (Hagger and Chatzisarantis, 2014; Sánchez-Oliva et al., 2015), allowing for this motivation to also improve educational values, such as social responsibility (Bagøien et al., 2010), as well as BPNs (Vasconcellos et al., 2020). Therefore, we can conclude that promoting motivation in Physical Education classes must begin from the earliest age to maintain these values in adolescence and at the end of the Secondary Education stage, when the values of physical activity and satisfaction of BPNs are low. The same is indicated by Abdoshahi et al. (2022), where Primary Education students have the highest levels of intrinsic motivation, autonomy support perception, and intention to be physically active.

Finally, regarding the third hypothesis, we did not find clear results, since we did not find statistically significant differences between boys and girls, neither in the distribution of the profiles, considering the standardized residuals, nor in the general values without taking the profiles into account. This contrasts with studies such as Aznar-Ballesta and Vernetta (2023), where girls were shown to have higher sports dropout rates than boys. Similarly, the review by Turhan (2020) indicates that girls tend to have higher values of intrinsic motivation and boys experience motivation more related to external rewards (Bugler et al., 2015). In turn, regarding the level of physical activity, Bugler et al. (2015) indicated that girls tend to perform less physical activity than boys, but in our case, this data is not indicated as it is similar in both genders. Likewise, Shen (2015) shows that motivation is usually higher in boys, and they have a greater perception of autonomy from their teachers, a result similar to that of Abdoshahi et al. (2022). On the other hand, we corroborate the results of Lauderdale et al. (2015), where it is indicated that when there is greater motivation (especially intrinsic), the levels of physical activity are higher. This may explain the non-existence of differences between genders in the intention to be physically active, since no differences were seen at the motivational level, showing the importance of generating an adequate climate of motivation to achieve these results. Therefore, we conclude in this sense that it is still necessary to continue investigating the role of gender in motivation and experiences related to SDT and the variables of responsibility, autonomy support, and the intention to be physically active. We also consider the necessity of following Turhan’s suggestions, which insist on the importance of studying the motivation of girls and boys, especially in the change of educational stage from Primary to Secondary Education, since the habits that are generated in the early stages are necessary to create suitable habits in the future. It is worth highlighting the study of Cerro-Herrero et al. (2022) and the necessity to implement interventions that promote the vision of active movement to take advantage of the high levels of intention to be physically active. The study conducted by Ahmadi et al. (2023) is also interesting as they identified 57 motivational behaviors of teachers that could explain most of the motivational behaviors of students.

## 5. Limitations and future research

The main limitations of the study indicate the cross-sectional nature of the study, which does not allow for cause-effect relationships. Another limitation is the sample obtained in Non-compulsory Education students, which was reduced in relation to the rest of the participants. In turn, the solution of four motivational profiles has been widely studied, but it could have been considered to carry out profile analysis including BPNs or another variable, which could have varied the solutions. Finally, the use of larger samples from other countries or samples consisting of students with different socio-economic characteristics could be interesting to investigate the results in different social and cultural centers.

As a future line of study, it is recommended to carry out intervention studies where motivation is promoted within Physical Education classes to improve the satisfaction of basic psychological needs, especially seeking to use a teaching style where autonomy is encouraged to also improve adherence to physical activity. On the other hand, carrying out longitudinal studies or including larger samples, including different contexts, would be of great interest to the scientific community. Finally, it would be interesting to expand the sample to university students in order to understand whether the reduction of the variables studied continues at this stage, with the inclusion of new variables like satisfaction due to the mediating effect following SDT and physical activity intention (Pérez-Quero et al., 2023).

## 6. Conclusions and practical applications

It is concluded that there were four motivational profiles called “high quantity,” “high quality,” “low quantity,” and “low quality,” which were related to each other with the psychological needs of autonomy, competence and social relationships, responsibility, autonomy support, and the intention to be physically active. All profiles have statistically significant differences between all variables.

The profile that had more appropriate values was the so-called “high quantity” profile, which makes it necessary to promote motivation in Physical Education students (including external motivation), especially from an early age, to improve adherence to physical activity and the promotion of educational values. On the other hand, “high quality” was the second profile with more positive values of the variables under study, and “low quality” was the profile with the lowest values in intention to be physically active, responsibility, satisfaction of basic psychological needs, and perception of support for autonomy. No differences were found between girls and boys, but the students in Primary Education were in the most self-determined profiles.

For these reasons, motivation should be emphasized following the SDT from an early age to generate better habits related to physical activity and greater responsibility. Similarly, the interventions carried out in the field of education should focus on promoting physical activity and values such as responsibility with a teacher who conducts their classes promoting autonomy, competence, and relatedness, following recommendations for the use of behaviors based on the SDT, such as those made by Ahmadi et al. (2023).

## Data availability statement

The raw data supporting the conclusions of this article will be made available by the authors, without undue reservation.

## Ethics statement

The studies involving humans were approved by University of Murcia Ethical Committee (1685/2017). The studies were conducted in accordance with the local legislation and institutional requirements. Written informed consent for participation in this study was provided by the participants' legal guardians/next of kin. Written informed consent was obtained from the individual(s) for the publication of any potentially identifiable images or data included in this article.

## Author contributions

DM-S: Writing – original draft, Writing – review & editing.

## Funding

The author(s) declare that no financial support was received for the research, authorship, and/or publication of this article.

## Conflict of interest

The author declares that the research was conducted in the absence of any commercial or financial relationships that could be construed as a potential conflict of interest.

## Publisher’s note

All claims expressed in this article are solely those of the authors and do not necessarily represent those of their affiliated organizations, or those of the publisher, the editors and the reviewers. Any product that may be evaluated in this article, or claim that may be made by its manufacturer, is not guaranteed or endorsed by the publisher.

## Footnotes

^1^ https://forms.gle/gbZMXMRBnrwnztUg7
